# Simultaneous Extraction and Depolymerization of Fucoidan from *Sargassum muticum* in Aqueous Media

**DOI:** 10.3390/md11114612

**Published:** 2013-11-21

**Authors:** Elena M. Balboa, Sandra Rivas, Andrés Moure, Herminia Domínguez, Juan Carlos Parajó

**Affiliations:** 1 Department of Chemical Engineering, University of Vigo (Campus Ourense), Polytechnical Building, As Lagoas, Ourense 32004, Spain; E-Mails: elenamba@uvigo.es (E.M.B.); sandrarivas@uvigo.es (S.R.); amoure@uvigo.es (A.M.); jcparajo@uvigo.es (J.C.P.); 2 Research Transfer and Innovation Centre (CITI), University of Vigo, Tecnopole, Rúa Galicia n° 2, Ourense 32900, Spain

**Keywords:** *Sargassum muticum*, hydrothermal treatment, alginate, fucoidan

## Abstract

The biomass components of the invasive seaweed *Sargassum muticum* were fractionated to allow their separate valorization. *S. muticum* (Sm) and the solid residue remaining after alginate extraction of this seaweed (AESm) were processed with hot, compressed water (hydrothermal processing) to assess the effects of temperature on fucoidan solubilization. Fucose-containing oligosaccharides were identified as reaction products. Operating under optimal conditions (170 °C), up to 62 and 85 wt% of the dry mass of Sm and AESm were solubilized, respectively. The reaction media were subjected to precipitation, nanofiltration and freeze-drying. The dried products contained 50% and 85% of the fucoidan present in Sm and AESm, respectively; together with other components such as phenolics and inorganic components. The saccharidic fraction, accounting for up to 35% of the dried extracts, contained fucose as the main sugar, and also galactose, xylose, glucose and mannose. The concentrates were characterized for antioxidant activity using the TEAC assay.

## 1. Introduction

*Sargassum muticum* (Yendo) Fensholt is an invasive macroalga living on the Western coasts that causes negative impacts on ecology, fishing and recreational activities. The unsuccessful eradication trials suggest a possible valorization of this alga biomass. The potential use of the whole alga as a low cost adsorbent for heavy metals’ removal was confirmed [1]. Also, *S. muticum* has been used as a substrate for hydrothermal processing, yielding a soluble fraction with antioxidant activity and a solid residue with potential as a fertilizer [2].

Marine brown seaweeds contain alginate, laminaran and sulfated polysaccharides known as fucoidans. Fucoidans are made up of fucose, galactose, mannose, xylose, glucose, uronic acids, sulfate substituents, and acetyl groups; and may also contain some protein components. Algal fucoidans are highly heterogeneous in structure, oppositely to the fucan sulfates from marine invertebrates, which are composed mainly of sulfated l-fucose and present a more simple and regular structure. Fucoidans may differ considerably in composition, molecular mass and structure, depending on the algal species considered [3], geographic location, environmental conditions, harvest season, vegetative and generative life-stages [4] or on the type of tissues sampled [5]. On the other hand, the extraction and purification conditions may affect the polysaccharide composition and structure of the isolated compounds (including charge density, distribution, degree of sulfation, molecular mass and linkage pattern) [6,7].

Since fucoidans can interfere with molecular mechanisms of cell-to-cell recognition, they are potent blockers of some biological processes, showing a variety of activities, including antiviral, antiinflammatory, antiangiogenic, antiproliferative, antitumoral, anticoagulant, immunomodulating and antioxidant properties [4,8,9,10]. Due to their non-toxic character, fucoidans have been recently explored for medicinal properties, but their detailed structural analysis is complex [11], and the structure–activity interrelationships are not yet clear [6,12].

*Sargassum* sp. is a potential source of fucose-containing sulfated polysaccharides [6], whose biological properties have received attention recently [13]. Some brown seaweed fucoidans have a backbone of 3-linked α-l-fucopyranose, whereas in other cases the backbone presents alternating 3- and 4-linked α-l-fucopyranose residues and sulfated galactofucans [11]. These latter compounds are prominently found in various *Sargassum* species [14], and are mainly built of (1→6)-β-d-galactose and/or (1→2)-β-d-mannose units with branching points formed by (1→3) and/or (1→4)-α-l-fucose, (1→4)-α-d-glucuronic acid, terminal β-d-xylose and sometimes (1→4)-α-d-glucose [14].

Crude fucoidans have been extracted from brown algae with water [14], diluted acid [4], diluted alkali, and 2% aqueous calcium chloride [3] under mild conditions. The extraction conditions have to be controlled carefully, as fucoidans are sensitive to degradation [6]. Since some methods cannot analyze large molecules, partial depolymerization of fucoidans has been carried out with acids [4] or radicals [12] before analysis. Low-chemical processes have been proposed for isolating pure, native fucoidans [7,9,10] in order to obtain products with enhanced biological activities [7,12].

Hydrothermal processing (also called autohydrolysis) is an autocatalyzed reaction suitable for the fractionation of vegetal biomass [15,16,17] that has been applied to alginate [18] and for algae fractionation, looking at the manufacture of multisulfated oligosaccharides [3] with structural features similar to the ones of the parent polymer [4].

This study provides an assessment on the aqueous processing of raw and alginate extracted *Sargassum muticum* biomass, with an emphasis on the sugar composition of the solubilized fraction. The effects of maximal temperature achieved in non-isothermal treatments on the fucoidan extraction yield, as well as on both composition and radical scavenging properties of the resulting products were measured.

## 2. Results and Discussion

**Figure 1:**
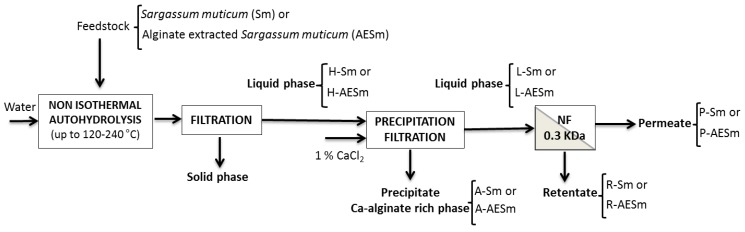
Flow diagram for the processing of *Sargassum muticum* (Sm) and alginate-extracted *S. muticum* (AESm).

The extraction methodology and conditions affect the composition, structure and size of the fucoidan fractions, which determine their functional properties and bioactivity. Alginate is one of the major components of *Sargassum* sp. [19,20], for which biological activities have been reported [21]. The proximate composition of the algal biomass (Sm) and the corresponding alginate-depleted product (AESm) are summarized in Table 1. Sm contained 26% minerals, 7% protein, 26% saccharides, and 20% acid insoluble residue. After removing alginate and extractives, the proportion of the other components in the AESm increased correspondently. Considering the yields of the process [2], 3.52 g fucose were present in 88.8 g Sm (d. b.), but only 2.29 g fucose were recovered in AESm. Crude fucoidan accounted for 4 wt%, in comparison with less than 1 wt% reported for *Sargassum* sp. [20], 8 wt% for *S. muticum* [19], and 7–9 wt% for sterile and reproductive tissues of *S. pallidum* [5]. The streams and products obtained during processing of Sm and AESm are indicated in the flow diagram of Figure 1.

**Table 1:** Composition of the raw materials used in this study.

Component	Content (wt%)	
	Sm	AESm
Glucose	8.41 ± 0.19	12.15 ± 0.08
Xylose	0.88 ± 0.05	0.78 ± 0.02
Galactose	2.52 ± 0.11	2.13 ± 0.07
Mannose	0.33 ± 0.02	0.42 ± 0.04
Fucose	3.97 ± 0.07	4.02 ± 0.14
AIR ^1^	20.28 ± 0.18	32.72 ± 3.79
Acetyl groups	0.22 ± 0.01	0.26 ± 0.02
Alginate	10.23 ± 0.75	
Protein	6.92 ± 0.08	8.38 ± 0.04
Ash	26.04 ± 0.07	30.66 ± 1.49
Total phenolics ^2^	1.03 ± 0.11	
Extractives		
96% Ethanol	6.32 ± 0.24	0.16 ± 0.01
Ethyl acetate	5.88 ± 0.27	1.42 ± 0.09
Hexane	4.10 ± 0.99	2.39 ± 0.08

^1^ AIR: acid insoluble residue; ^2^ Determined in the ethanolic extract and expressed as gallic acid equivalents.

**Figure 2:**
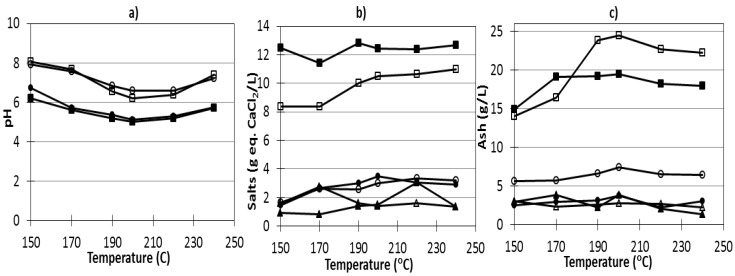
Effect of autohydrolysis temperature on the (**a**) pH, (**b**) salt content, and (**c**) ash content in samples from the streams (●) H-Sm, (○) H-AESm, (■) L-Sm, (□) L-AESm and (▲) R-Sm and (△) R-AESm generated according to the scheme for processing *Sargassum muticum* (Sm) and alginate extracted *S. muticum* (AESm) in Figure 1.

The effects of temperature on the pH of the reaction media is shown in Figure 2a. The pH of AESm-containing media was higher than that obtained with Sm. In both cases, the minimum pH was reached operating at 200–210 °C, and a slight decrease after CaCl_2_ addition was observed. The lower pH attained after processing the whole algae could be ascribed to the decomposition of alginate, as the hydrothermal treatment of alginate results in the formation of water soluble acids [18]. The salt concentrations are shown in Figure 2b. A maximum of 12 g CaCl_2_ equivalents/L was found for liquors from Sm processing (stream L-Sm in Figure 1), whereas a steady increase from 8 to 11 g/L was observed when the media made with AESm were heated from 170 to 240 °C (stream L-AESm in Figure 1). Both types of reaction media showed higher salt concentrations when the autohydrolysis temperature increased, whereas slightly lower values were observed for the diafiltration retentates. The ash content varied slightly with the treatment temperature, being higher in L-Sm and in L-AESm than in H-Sm, H-AESm, R-Sm and R-AESm (Figure 2c).

**Figure 3:**
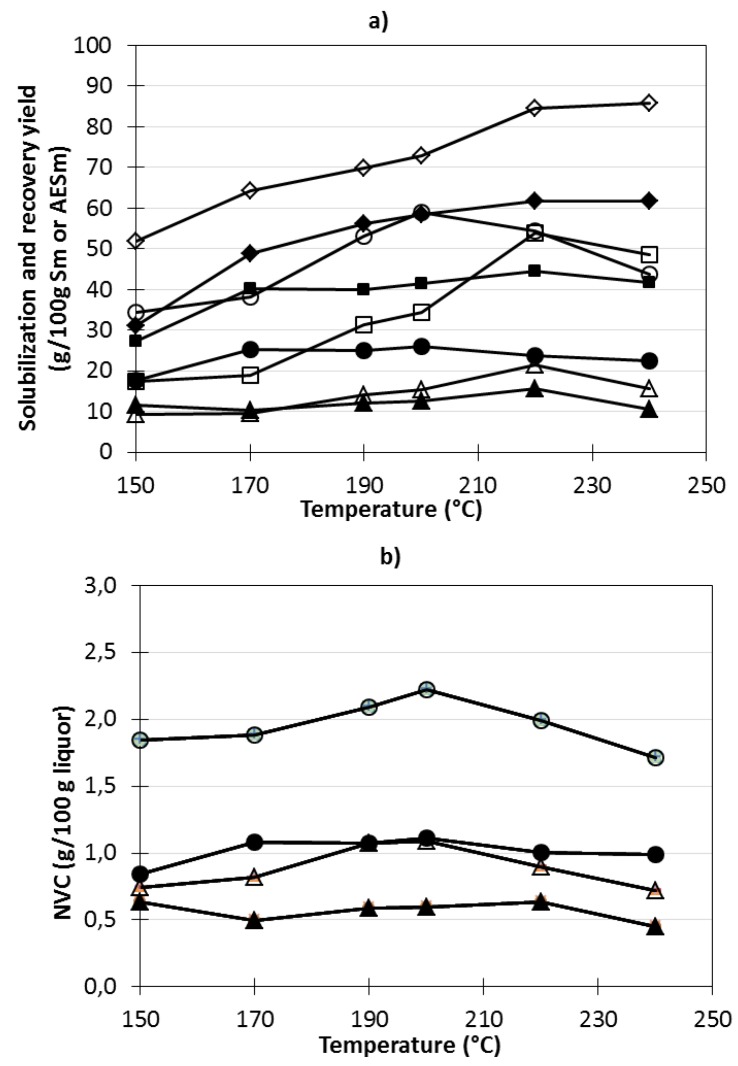
Effect of temperature on (**a**) the solubilization of Sm and AESm in the (♦) H-Sm and (◊) H-AESm streams and on the recovery of solutes in the liquors generated from processing of Sm and AESm: (●) H-Sm, (■) L-Sm and (▲) R-Sm streams (g Sm extract/100 g Sm) and (○) H-AESm, (□) L-AESm and (△) R-AESm streams (g AESm extract/100 g AESm), and on (**b**) the generation of non-volatile components (NVC) (g NVC/100 g liquor) found in streams (●) H-Sm and (○) H-AESm and in (▲) R-Sm and (△) R-AESm.

Figure 3 shows the influence of temperature on the total solubilization yield, on the non-volatile components (NVC) of streams H-Sm and H-AESm, on the recovery yields in streams H, L and R for Sm and AESm samples and on the NVC content of the liquors obtained from these streams after CaCl_2_ precipitation, L-Sm and L-AESm and after diafiltration, R-Sm and R-AESm. The amount of solubilized products in streams H-Sm and H-AESm (measured as percentages with respect to the dry mass of materials subjected to hydrothermal processing) increased with temperatures in the range studied, reaching values of up to 61 wt% at 220–240 °C for Sm and 86 wt% for AESm. In this latter case, the overall extraction yield obtained in the process (including alginate removal and hydrothermal processing) was close to 97%. The extract content of NVC varied slightly with extraction temperature. The extracts with the highest NVC contents were obtained in liquors from AESm treatments.

**Figure 4:**
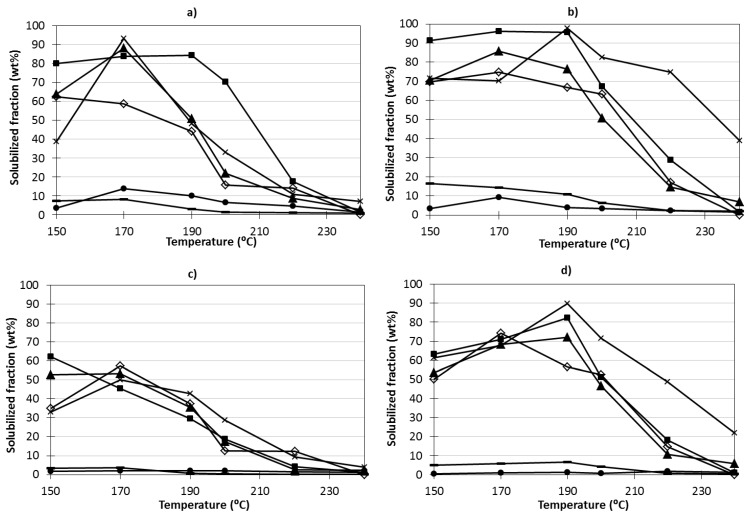
Effects of temperature on the recovery percentage of structural components of saccharides in the liquors from autohydrolysis (**a**) H-Sm and (**b**) H-AESm and in purified streams (**c**) R-Sm and (**d**) R-AESm, expressed as percentage of the respective amounts present in the starting materials (Sm or AESm). Percentage conversion to soluble products of (▲) fucose, (X) galactose, (■) xylose, (●) glucose, (◊) mannose and (**^_^**) uronic acids.

The extraction percentages of the various constituent sugars, measured in the streams H-Sm and H-AESm, in respect to the amount of the corresponding units of the starting materials (Sm or AESm) are shown in Figure 4. The same figure presents the data concerning the recovery percentages in the streams obtained after precipitation and diafiltration (R-Sm and R-AESm). The maximum recovery of fucose units in soluble products H-Sm and H-AESm occurred at 170 °C, accounting for 85.6%–88.0% of the amount present in substrates. Considering the recovery values in streams R-Sm and R-AESm, up to 55%–72% was obtained. Higher recovery of xylose and galactose structural units in the soluble products was observed, with values higher than 90%. At temperatures above 200 °C, the recovery of sugars forming part of the soluble saccharides decreased markedly. In all experiments, the recoveries of glucosyl, mannosyl and uronyl units in oligosaccharides were limited, no matter the operational conditions and type of substrates.

In a previous study, the temperature range 190–200 °C was identified as optimal for AESm processing, based on the results achieved for total yield, yield in phenolics and ABTS radical scavenging capacity of isolates [2]. The solubilization yields decreased with temperatures in the range tested, a fact ascribed to the instability of fucose-containing compounds under harsh conditions [6].

Similar fucoidan extraction yields have been reported for mild acid treatments of other *Sargassum* sp. In most cases, the algae were extracted with organic solvents to remove pigments and phenol compounds before acidic processing. Fucose-containing polysaccharides were obtained at 5.2% yield from *S. horneri* subjected to acid extraction [22], at 5% yield from *Sargassum* *trichophyllum* refluxed with water [10], and at 7%–9% yield from *Sargassum* sp. [6] and *S. pallidum* [5]. The reported fucoidan extraction yields for other brown algae lie in a wider range than for *Sargasssum* sp. (up to 20% for *Pelvetia* or to 11% for *Ascophyllum nodosum*) [9].

The recovery of uronyl groups in oligosaccharides was higher in experiments with AESm than in assays with Sm. Under typical conditions for hydrothermal processing, the alginate structural units (mannuronic and guluronic acid), are much more reactive than cellulose and cellulose-derived saccharides, due to their hydrophilic character and the self-catalytic nature of carboxylic groups. Alginate decomposition has been proposed to proceed through an acid hydrolysis pathway at 22–80 °C, influenced by the presence of phenolic compounds susceptible to oxidation, transition metal ions, oxygen and the pH of the solution [23]. Under typical conditions for hydrothermal processing [18] and under subcritical conditions, very short timeframes are required to decompose alginate [24].

**Figure 5:**
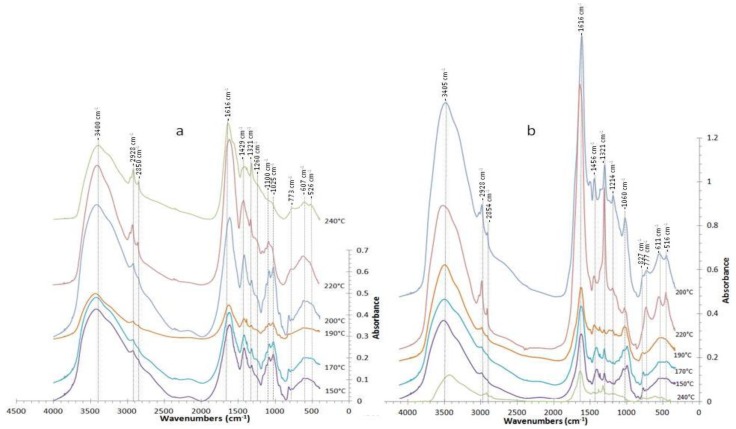
FT-IR profiles of the alginate rich fractions from (**a**) A-Sm and (**b**) A-AESm extracted and recovered according to the scheme in Figure 1.

Under non isothermal processing (180–240 °C) in an inert atmosphere, alginate depolymerized into oligosaccharides, monosaccharides and decomposition products (lactic acid and glycolic acid and low molecular weight products). The decomposition of alginate is promoted by temperature and probably took place by releasing mannuronic acid at short reaction times, followed by the release of guluronic acid. Mannuronic acid is rapidly decomposed into water soluble acids, solids and gas [18]. Uronic acids were identified as the most abundant structural units of the soluble saccharides. Both fucoidans and residual alginate contain uronic acids as structural units. This behaviour was confirmed in the Fourier transform infrared spectroscopy (FT-IR) spectra of the alginate rich fraction A-Sm (Figure 5), with two IR bands at approximately 1100 and 1025 cm^−1^, assigned to mannuronic and guluronic units, respectively [8,21]. The presence of a strong band at 1025 cm^−1^ and a small shoulder at 1080–1100 cm^−1^ in samples from Sm treatments at 170 or 190 °C has been ascribed to the presence of small amounts of mannuronic acid in the homoguluronan-enriched alginate fraction [21]. No signal appeared at 1025 cm^−1^ in AESm processed at temperatures above 200 °C; whereas a progressive darkening of the media was observed, as reported for hydrothermally treated alginate solutions [18].

The FT-IR spectra of the alginate rich products, A-Sm and A-AESm, presented characteristic bands at 2923.7–2928.9 cm^−1^ (C–H stretching), 1616.7–1652.5 cm^−1^ (carbohydrate O–C–O asymmetric stretching vibrations), 1419.1–1429.6 cm^−1^ (C–OH deformation vibration), 1321.3–1326.3 cm^−1^ (C–C–H, and O–C–H deformation), 1074.2–1083.4 cm^−1^ (C–O stretching vibrations) and 1033 cm^−1^ (C–O and C–C stretching vibrations) of pyranose rings, at 946 cm^−1^ (C–O stretching vibration uronic acid), 900 (α-l-gulopyranuronic asymmetric ring vibration) and 815 cm^−1^ (mannuronic residues) [25].

Bands were observed at 821–823 cm^−1^ for A-Sm samples and at 816–820 cm^−1^ for A-AESm at temperatures below 200 °C. These bands have been ascribed to mannuronic acid, although sulfation of an equatorial primary hydroxyl group attached to the pyranose ring of hexoses shows an absorption band at 820 cm^−1^, attributable to the C–O–S vibration of equatorial sulfate groups [3,26,27]. Samples from Sm and from AESm showed shoulders or small shoulders at 1257.4–1263.4 cm^−1^, at the characteristic band of sulfate groups in polysaccharides, corresponding to the S=O stretching vibration of the sulfate group common to all the sulfate esters bands between the regions 1240–1272 cm^−1^ [3,14,25,26]. Some of these samples presented a very light band close to 622 and 583 cm^−1^, attributed to the asymmetric and symmetric O=S=O deformation of sulfates [28].

**Figure 6:**
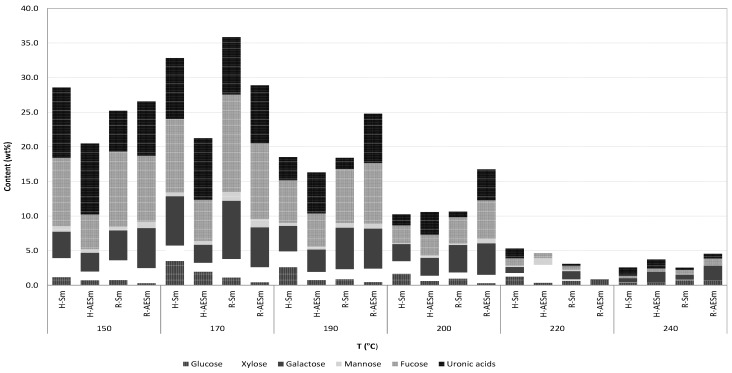
Relative proportions of sugars making part of soluble saccharides obtained after drying (H-Sm and H-AESm) and after CaCl_2_ precipitation and diafiltration (R-Sm and R-AESm), expressed as weight percentage.

The refined products obtained from streams R-Sm and R-AESm by precipitation with calcium chloride and diafiltration were freeze-dried and characterized. HPLC analysis showed the presence of structural units of saccharides containing fucose, galactose, xylose, glucuronic acid and mannose, in agreement with fucan or heterofucan parent polymers. Monosaccharides were not found in the liquors from hydrothermal processing of AESm and Sm. The relative amount of the structural units making part of the saccharides obtained at the various treatment temperatures are presented in Figure 6 as a function of the treatment temperature. As a general pattern, the maximal concentrations of constituent sugars were achieved in treatments performed under low- or medium-severity conditions, harsher autohydrolysis conditions resulted in decreased amounts of soluble saccharides. At temperatures above 200 °C, the degradation of sugars was evident and the saccharidic fraction accounted for less than 10% of the solubilized fraction. Fucose was the major structural component of soluble saccharides, except in H-AESm, followed by uronic acids and galactose. Galactose content was lower in the H-AESm streams. Operating at the optimal temperature (170 °C), the soluble saccharides accounted for 30%–35% of the dried extracts, and the product showed a mass ratio of the constituent sugars fucose:galactose:glucose:xylose:mannose of 1:0.67:0.33:0.21:0.06 for H-Sm and 1:0.44:0.33:0.22:0.09 for H-AESm, 1:0.60:0.08:0.19:0.09 for R-Sm and 1:0.53:0.04:0.20:0.11 for R-AESm.

Related values have been reported for extracts from *Sargassum* sp. For example, a fucoidan from *S*. *stenophylum* obtained by extraction with water and purified by ethanol and cetylpyridinium chloride precipitation was made up of fucose, galactose and xylose, with smaller amounts of mannose, glucose and glucuronic acid [14]. Fucose and galactose were the most abundant constituents of a fucoidan from *S. trichophyllum* [10] and also in the crude extract isolated from the reproductive tissue of *S. pallidum* by acidic processing and dialysis [5], whereas a crude *S. tenerrimum* fucoidan containing fucose, xylose, galactose and glucose was isolated by acidic processing, dialysis and freeze-drying [21]. *Sargassum filipendula* heterofucans obtained by proteolytic digestion and acetone precipitation were composed of fucose, glucose, glucuronic acid, galactose and sulfate; in some fractions mannose and glucuronic acids were not detected [13]. Rhamnose, polyphenols and low amounts of xylose were also found in polysaccharides from *Sargassum* sp. obtained by ethanol extraction followed by acidic processing, concentration, dialysis, and fractionation [20]. Heterofucans isolated from other *Sargassum* sp. were composed of fucose, galactose, xylose, glucuronic acid and mannose [29].

Owing to the resistance of glucuronic acid to hydrolytic degradation, the products extracted at short reaction times presented comparatively high fucose contents, whereas longer times resulted in increased extraction yields, but also in products with lower contents of fucose and sulfate and higher proportions of glucuronic acid [6,14]. It has been suggested that the fraction resistant to acidic hydrolysis present in brown algae contained a core of glucuronic acid and mannose residues [4]. Some brown seaweed sulfated polysaccharides have a backbone consisting of 3- linked α-l-fucopyranose, while in others, as in *Sargassum* species, the backbone is made up of alternating 3- and 4-linked α-l-fucopyranose residues and sulfated galactofucans [11,14].

**Figure 7:**
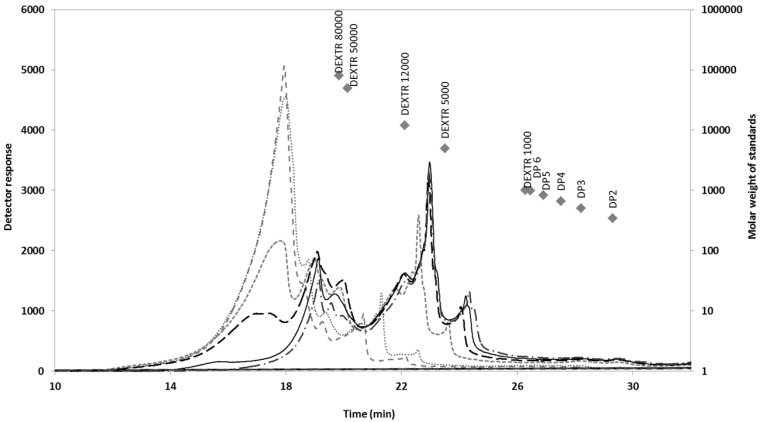
HPSEC elution profiles determined for the samples R-Sm and R-AESm. The MW (♦) of dextran standards are also presented. (- - -) 150 °C, (**—**) 170 °C, (-----) 190 °C, (**····**) 200 °C, (**—** ) 220 °C and (− ^.^ − ^.^) 240 °C.

Figure 7 shows the HPSEC profiles determined for standards and retentates from hydrothermal liquors. Expectedly, higher temperatures resulted in increased depolymerization of the polysaccharides, generating polymers with molecular masses higher than 80 kDa. Low molecular weight polymers with molar masses in the range 5–12 kDa were obtained in experiments at 190 °C or higher. In literature, low temperature operation (30–50 °C) led to fractions with molar mass ≥10 kDa after 72 h of treatment, whereas severer conditions could cause degradation [12,30]. The production of fucoidans at 30 °C and pH 3 in media containing 0.2 M H_2_O_2_ resulted in the production of compounds with molar masses above 100 kDa after 6 h, which were reduced to approximately 10 kDa after 72 h. In comparison, the molar masses of the products obtained at 90 °C for 4 h were below 1 kDa [12].

The biological properties of fucoidans depend on a number of factors, including the relative abundance of sulfate groups, structural features, and molar mass distribution. As fucoidans must permeate the biological membranes to exert their effects *in vivo*, depolymerization of high molar mass compounds to fragments below 30 kDa results in more active fractions [7,12]. Low-molecular-weight, sulfated polysaccharides are effective radical scavengers [12], whereas compounds of 26–35 kDa from *S. tenerrimum* and *S. plagiophyllum* showed activity as antiviral agents and as inhibitors of carcinogen metabolic activation compounds, respectively [21].

A number of properties have been reported for polysaccharides from *Sargassum* sp., including reducing, chelating, and scavenging activities against DPPH, hydroxyl and superoxide radicals [13,29]. The antioxidant action of samples from autohydrolysis of *S. muticum* were evaluated in this work by measuring their scavenging capacity against the ABTS radical, and expressed as Trolox equivalent antioxidant capacity (TEAC assay). This method was selected due to its operational simplicity, and because the TEAC values of related compounds are available. Additionally, the results of this assay often correlates with the ones of the methods FRAP, DPPH and Folin, due to similarities in the chemical fundament. The ABTS radical scavenging capacity of the extracts, higher for H-Sm and L-Sm samples, increased with temperature in the studied range (Table 2), in agreement with data reported for non diafiltered samples [2]. The antioxidant activity of the target products was moderate under all the operational conditions. Typically, one gram of the extracts was equivalent to less than 0.25 g of Trolox. Related results have been reported for crude fucoidans and fractions [13]. The antioxidant activity of the crude extracts can be ascribed to the effects of different compounds, including phenolic compounds and fucoidan. Both the phenolic extraction yield and the content of the extracts, measured as gallic acid equivalents, showed a steady increase from 170 to 220 °C, whereas the sugar and fucoidan content decreased progressively with the temperature of hydrothermal processing (Figure 6). The total phenolic contents of extracts coming from the nanofiltration (streams R-Sm and R-AESm) showed maximum values at 190 °C, with up to 6% for extracts from AESm. Higher values were reported for the H-AESm stream [2], suggesting that some small phenolic components could have permeated the membrane.

**Table 2:** (**a**) Phenolic extraction yield of H-Sm, L-Sm and R-Sm streams (g GAE/100 g Sm) and of H-AESm, L-AESm and R-AESm streams (g GAE/100 g AESm); (**b**) phenolic content of extracts (g GAE/100 g extract); and (**c**) TEAC value (g Trolox equivalents/g extract), for H-Sm, L-Sm, R-Sm, H-AESm, L-AESm and R-AESm extracts.

	H-Sm			H-AESm			L-Sm			L-AESm			R-Sm			R-AESm		
T (°C)	a	b	c	a	b	c	a	b	c	a	b	c	a	b	c	a	b	c
150	1.13	6.42	0.18	1.58	4.61	0.12	0.71	2.59	0.06	0.34	1.95	0.05	1.39	11.94	0.10	0.26	2.84	-
170	1.49	5.87	0.14	2.04	5.35	0.13	1.30	3.24	0.07	0.44	2.30	0.05	-	-	-	0.25	2.60	0.06
190	1.87	7.52	0.18	3.59	6.76	0.16	1.65	4.14	0.09	1.31	4.20	0.10	-	-	-	0.84	6.01	0.15
200	2.34	9.00	0.21	3.05	5.17	0.22	1.92	4.65	0.10	1.72	5.01	0.11	0.74	5.86	0.10	0.84	5.49	0.15
220	2.31	9.73	0.23	3.10	5.69	0.26	2.24	5.04	0.12	2.76	5.13	0.11	1.42	9.15	0.14	1.28	5.98	0.16
240	2.19	9.76	0.24	2.96	6.78	0.24	2.13	5.12	0.11	2.00	4.13	0.09	0.80	7.71	0.19	0.89	5.64	0.13

a: g GAE/100 g dried Sm; b: g GAE/100 g extract; c: g Trolox/g extract.

## 3. Experimental Section

### 3.1. Materials

*Sargassum muticum* specimens were collected manually in Praia da Mourisca (Pontevedra, Spain) in June 2010 and stored at −18 °C. This season was selected because in summer the biomass contains maximal amounts of phenolics and fucoidan [19]. Algal material was defrosted, cleaned, washed with tap water, oven-dried at 50 °C, and ground.

### 3.2. Processing Methods

#### 3.2.1. Alginate Extraction

Ground *S. muticum* samples were contacted with 1% formaldehyde, and the resulting solids were subjected to three consecutive stages of water washing, 0.2 N sulphuric acid extraction at room temperature for 4 h, three stages of water washing and 1% sodium carbonate extraction at room temperature for 15 h, using in all stages a liquid:solid ratio of 50 (v/w, wet basis). Alginate was recovered from the soluble fraction by ethanol precipitation, yielding alginate-free solids (AESm), which were assayed as substrates for aqueous processing in this work. This material was oven-dried at 50 °C and stored in sealed bags until use.

#### 3.2.2. Treatments with Hot Compressed Water

Both the ground dried *S. muticum* (Sm) or AESm were mixed with water at a liquid:solid ratio of 30:1 (w/w, dry basis) and heated in a stainless steel reactor (Parr Instr. Co., Moline, IL, USA) reaching temperatures in the range of 150–240 °C. Once the target temperature was reached, the reactor was cooled immediately, and the liquid and solid phases were separated by filtration. The residual alginate in liquid phase was precipitated by adding 1% CaCl_2_, and the supernatant was diafiltered through a 0.3 kDa membrane to remove salts. The retentate was freeze-dried until analysis. The scheme of this process is shown in Figure 1.

### 3.3. Analytical Methods

Samples of liquors were filtered through 0.45 µm cellulose acetate membranes, neutralized with barium carbonate, and assayed by HPLC for glucose, xylose, mannose and galactose using a 1100 series Hewlett-Packard chromatograph fitted with a refractive index detector (operating at 50 °C) and a 300 × 7.8 mm CARBOsep CHO 682 column (Transgenomic, Glasgow, UK) operating at 80 °C. Distilled water was used as the mobile phase (flow rate, 0.4 mL/min). Fucose and acetic acid were determined by HPLC-RI using the same instrument and a 300 × 7.8 mm Aminex HPX-87H column (BioRad, Hercules, CA, USA) operating at 60 °C (mobile phase: 0.003 M H_2_SO_4_, flow rate: 0.6 mL/min). The concentrations of saccharides and linked acetyl groups were determined from the concentrations of monosaccharides and acetic acid present in samples previously subjected to a quantitative posthydrolysis (treatment with 4% sulfuric acid at 121 °C for 20 min). Before analysis on a CARBOsep CHO 682 column, posthydrolysis samples were neutralized with barium carbonate.

Uronic groups were determined spectrophotometrically by the method of Blumenkrantz and Asboe-Hansen [31], using galacturonic acid as a standard for quantitation.

The content of non-volatile compounds (NVCs) in the liquors was measured by oven-drying at 105 °C until constant weight.

Conductivity was measured in a HI 8633 Hanna instrument (Guipúzcoa, Spain), and the salt concentrations were expressed in terms of CaCl_2_ equivalents.

Ash was determined by calcination at 575 °C. The total phenolic content was measured by the Folin-Ciocalteu method [32], and expressed as gallic acid equivalents (GAE). All analyses were performed at least in triplicate, and the results are reported on oven-dry mass basis. The protein content was determined from the total Kjeldhal nitrogen, using the 5.38 conversion factor for brown algae [33].

Freeze-dried alginate fractions were blended with KBr, and the mixture was dried with an infrared lamp for 10 min. A tablet was prepared with the mixture by using a press at 7 ton pressure. FTIR spectra were recorded in transmission mode in the wavelength range 400–4000 cm^−1^ with phase resolution 4 cm^−1^, and averaging 32 scans min^−1^ using a FTIR Nicolet 6700, Thermo Scientific infrared spectrophotometer, equipped with an OMNIC software for data analysis with a DTGS detector, KBR beamsplitter and ETC-Ever Glo optical bench source.

#### High Performance Size Exclusion Chromatography (HPSEC)

The molar mass distribution of the target products was determined by HPSEC using two 300 × 7.8 mm TSKGel G3000PWXL columns in series (Tosoh Bioscience, Stuttgart, Germany), in combination with a 40 × 6 mm PWX-guard column, operating at 30 °C with a refractive index detector. The mobile phase was distilled water (flow rate, 0.6 mL/min). Dextrans (1000–80,000 g/mol) from Fluka (parent company of Sigma-Aldrich, St. Louis, MO, USA) and oligosacharides in the range DP2-6 (Megazyme, Co. Wicklow, Ireland) were used as calibration standards.

## 4. Conclusions

The biological properties of fucoidans depend on a number of factors, including composition, structure, type of seaweed, extraction and sampling methods, and environmental and seasonal factors. The lack of both standardized protocols for the extraction and purification of products suitable for specific nutraceutical applications has boosted the research on new extraction and purification processes, with the intention of obtaining products keeping the structural features of the native polymers. Hot water processing under subcritical conditions is effective, environmentally friendly, and technologically suitable for fractionating *Sargassum* sp. biomass, enabling a simultaneous extraction and depolymerization of fucoidans in a single stage. Native biomass and alginate-depleted substrates were feedstocks suitable for hydrothermal processing. From 50% to 85% of the fucoidan contained in these two types of materials was solubilized and recovered after consecutive stages of alginate precipitation, diafiltration and freeze-drying. The dried extracts contained 30%–35% fucoidans, which were characterized for structural components, molar mass distribution and TEAC.
